# Low-Dose vs. Standard-Dose Intravenous Alteplase in Bridging Therapy Among Patients With Acute Ischemic Stroke: Experience From a Stroke Center in Vietnam

**DOI:** 10.3389/fneur.2021.653820

**Published:** 2021-04-09

**Authors:** Duy Ton Mai, Viet Phuong Dao, Van Chi Nguyen, Dang Luu Vu, Tien Dung Nguyen, Xuan Trung Vuong, Quoc Viet Bui, Ha Quan Phan, Quang Tho Pham, Hoang Kien Le, Anh Tuan Tran, Quang Anh Nguyen, Phuc Duc Dang, Hoang Nguyen, Hoang Thi Phan

**Affiliations:** ^1^Stroke Center, Bach Mai Hospital, Ha Noi, Vietnam; ^2^Department of Emergency and Intensive Care, Ha Noi Medical University, Ha Noi, Vietnam; ^3^Radiology Center, Bach Mai Hospital, Ha Noi, Vietnam; ^4^Stroke Department, The 103 Hospital, Ha Noi, Vietnam; ^5^Wicking Dementia Research and Education Centre, University of Tasmania, Hobart, TAS, Australia; ^6^College of Health and Medicine, Menzies Institute for Medical Research, University of Tasmania, Hobart, TAS, Australia

**Keywords:** acute ischemic stroke, mechanical thrombectomy, dose, bridging therapy, anterior large artery occlusion, alteplase, intravenous thrombolysis

## Abstract

**Background:** To date, the role of bridging intravenous thrombolysis before mechanical thrombectomy (MTE) is controversial but still recommended in eligible patients. Different doses of intravenous alteplase have been used for treating patients with acute ischemic stroke from large-vessel occlusion (LVO-AIS) in Asia, largely due to variations in the risks for intracerebral hemorrhage (ICH) and treatment affordability. Uncertainty exists over the potential benefits of treating low-dose alteplase, as opposed to standard-dose alteplase, prior to MTE among patients with LVO-AIS.

**Aim:** The aim of the study was to compare outcomes of low- vs. standard-dose of bridging intravenous alteplase before MTE among LVO-AIS patients.

**Methods:** We performed a retrospective analysis of LVO-AIS patients who were treated with either 0.6 mg/kg or 0.9 mg/kg alteplase prior to MTE at a stroke center in Northern Vietnam. Multivariable logistic regression models, accounting for potential confounding factors including comorbidities and clinical factors (e.g., stroke severity), were used to compare the outcomes between the two groups. Our primary outcome was functional independence at 90 days following stroke (modified Rankin score; mRS ≤ 2). Secondary outcomes included any ICH incidence, early neurological improvement, recanalization rate, and 90-day mortality.

**Results:** We analyzed data of 107 patients receiving bridging therapy, including 73 with low-dose and 34 with standard-dose alteplase before MTE. There were no statistically significant differences between the two groups in functional independence at 90 days (adjusted OR 1.02, 95% CI 0.29–3.52) after accounting for potential confounding factors. Compared to the standard-dose group, patients with low-dose alteplase before MTE had similar rates of successful recanalization, early neurological improvement, 90-day mortality, and ICH complications.

**Conclusion:** In the present study, patients with low-dose alteplase before MTE were found to achieve comparable clinical outcomes compared to those receiving standard-dose alteplase bridging with MTE. The findings suggest potential benefits of low-dose alteplase in bridging therapy for Asian populations, but this needs to be confirmed by further clinical trials.

## Introduction

The role of bridging thrombolysis before mechanical thrombectomy (MTE) has been controversial (1), but it is still recommended in eligible patients (1–4). In a recent meta-analysis of 38 observational studies, bridging therapy appears to be associated with improved functional independence without evidence for safety concerns, and this is compared to MTE for patients with acute ischemic stroke from large-vessel occlusion (LVO-AIS) (5). Randomized controlled trials that assessed whether primary MTE was non-inferior to the bridging strategy of intravenous thrombolysis (IVT) alteplase immediately followed by MTE in AIS patients presenting to thrombectomy capable centers produced mixed results. The SKIP (The Randomized Study of EVT With Vs. Without Intravenous Recombinant Tissue-Type Plasminogen Activator in Acute Stroke With ICA and M1 Occlusion) trial, which included patients with LVO-AIS in Japan, was unable to demonstrate the noninferiority of MTE alone over bridging therapy with low-dose alteplase before MTE (6). Conversely, the DIRECT-MT (Direct Intraarterial Thrombectomy in Order to Revascularize Acute Ischemic Stroke Patients With Large Vessel Occlusion Efficiently in Chinese Tertiary Hospitals) trial conducted in China showed that MTE alone was non-inferior (≤20% margin of confidence) to MTE preceded by standard-dose alteplase with regard to the primary outcome (90-day modified Rankin Scale shift) (7). The DEVT (Direct Endovascular Thrombectomy vs. Combined IVT and Endovascular Thrombectomy for Patients With Acute Large Vessel Occlusion in the Anterior Circulation) trial in China also demonstrated the non-inferiority of primary MTE treatment over the bridging therapy (standard-dose IVT + MTE) in functional independence (noninferiority margin of 10%) (8). However, there may be individual factors in the decision-making process that were not captured in the clinical trials (2). Accumulated trial data so far are insufficient to negate the value of alteplase bridging at thrombectomy capable centers (2), awaiting the results of ongoing trials including the DIRECT-SAFE (DIRECT Endovascular Clot Retrieval Vs. Standard Bridging Thrombolysis With Endovascular Clot Retrieval; NCT03494920) and MR CLEAN-NO IV: Intravenous treatment followed by intra-arterial treatment vs. direct intra-arterial treatment for acute ischemic stroke caused by a proximal intracranial occlusion (9).

In 2010, an open-label, nonrandomized, observational study suggested that low-dose (0.6 mg/kg) intravenous alteplase within 3 h of stroke onset could be safe and effective for the Japanese population (10). Various doses of intravenous alteplase have been used for treating patients with LVO-AIS in Asia, which is largely due to the reduced cost of low-dose IVT and its lower anticipated intracerebral hemorrhages (ICH) rates, compared to standard-dose IVT (11). Findings from the Enhanced Control of Hypertension and Thrombolysis Stroke Study (ENCHANTED) trial involving predominantly Asian patients, failed to prove the noninferiority of low-dose to standard-dose intravenous alteplase with respect to death and functional outcomes at 90 days, but there were fewer ICH in the patients receiving low-dose alteplase (6). Since the ENCHANTED study was published, there has been limited evidence comparing clinical outcomes between low- and standard-dose intravenous alteplase in Asian populations. Uncertainty exists over the potential benefits of treating low-dose alteplase, as opposed to standard-dose alteplase, prior to MTE among patients with LVO-AIS presenting directly to a thrombectomy-capable stroke center, particularly for Asian populations.

The aim of the study was to compare clinical outcomes of low-dose vs. standard-dose intravenous alteplase combined with MTE in patients with LVO-AIS.

## Materials and Methods

### Study Design

The study was conducted at the Stroke Center of Bach Mai Hospital, Hanoi, Vietnam. As one of the leading stroke centers nationally, we have been providing acute care to approximately 10,000 episodes of stroke each year. We retrospectively included patients with LVO-AIS at the Stroke center who received bridging therapy between 2017 and 2019. The included patients must meet the criteria for treatment of intravenous alteplase within a 4.5-h window from stroke onset as well as MTE within the 6-h window from stroke onset. We applied the inclusion and exclusion criteria for intravenous alteplase within 4.5 h of onset as recommended in the 2013 American Heart Association/American Stroke Association (AHA/ASA) Guidelines (10) [consistent with the updated 2018 AHA/ASA Guidelines (12)] and the 2015 AHA/ASA Scientific Rationale for the Inclusion and Exclusion Criteria for Intravenous Alteplase in Acute Ischemic Stroke (13). At Bach Mai hospital, we have also followed the inclusion and exclusion criteria for treating MTE within 6 h of stroke onset stated in the 2015 AHA/ASA Focused Update of the 2013 Guidelines for the Early Management of Patients With Acute Ischemic Stroke Regarding Endovascular Treatment (1). At the Stroke Center, bridging therapy was performed immediately following thrombolysis, and we continued alteplase infusion during thrombectomy even with those who achieved successful recanalization.

The Japanese drug safety authority has approved the use of low-dose alteplase after an open-label, nonrandomized, observational study showed that it could be safe and effective compared to standard-dose alteplase for the Japanese population (10). Given the concerns of intracerebral hemorrhage (ICH), treatment affordability, and lack of clinical evidence for the optimal dose for Vietnamese patients (10), we used low-dose alteplase for thrombolytic therapy before MTE among patients with LVO-AIS at the Stroke Center of Bach Mai Hospital from 2010 to 2018. We later used standard-dose alteplase in combination with thrombectomy for the eligible patients hospitalized in 2019, following the commencement of the ENCHANTED trial (6). Therefore, the patients included in this single-center retrospective study were from two time periods. The 1st period from 2017 to 2018 included the patients who received low-dose intravenous alteplase (0.6 mg/kg; 15% bolus and 85% as infusion over 1 hour), and the 2nd period in 2019 included those receiving standard-dose alteplase (0.9 mg/kg; 10% bolus, and 90% as infusion over 1 h).

All the included patients received computed tomography (CT) and computed tomography angiography (CTA) scans to decide if alteplase should be administered. The patients were immediately referred to the intervention room for MTE if the CTA revealed an anterior large artery occlusion. MTE was performed via a transfemoral approach with either SOLITAIRE (stent retriever system), PENUMBRA (aspiration system), or SOLUMBRA (aspiration plus extraction technique).

This retrospective study was approved by the Ethics Committee of Hanoi Medical University, No 187/HĐĐĐĐHYHN on February 20, 2016.

### Clinical Assessments and Measured Outcomes

Patients were assessed by clinicians and their data were obtained at 24, 72 h, 7 days (or at discharge if sooner), 28, and 90 days after hospital admission.

Clinical assessments were performed using the National Institute of Health Stroke Scale (NIHSS), modified Rankin scale (mRS), and brain imaging CT scan, and/ or magnetic resonance imaging [MRI] within 24 h of admission. Information on patient characteristics and care received in the hospital was obtained from the medical records. They included (1) socio-demographics: age, sex; (2) pre-stroke factors: pre-morbid mRS, use of medications, risk factors (body weight, blood pressure, and smoking), and comorbidities (e.g., history of stroke, hypertension, atrial fibrillation, diabetes mellitus, hypercholesterolemia); and (3) stroke-related clinical factors. The clinical factors included stroke severity (initial NIHSS), ischemic stroke subtype according to the Acute Stroke Treatment (TOAST) criteria (14), onset to hospital arrival, door to needle time, needle to groin puncture time, groin to recanalization time, intracranial atherosclerosis, Alberta stroke program early CT score (ASPECT) score (15), and occlusion sites [e.g., internal carotid artery, tandem, and middle cerebral artery (MCA) on cerebral angiography]. Other factors were the MTE technique performed, a need for placement of permanent intracranial stent during MTE to achieve recanalization, and the number of attempts to achieve recanalization. Where available, information on the collateral state was obtained from the medical records. The overall state of collaterals for each patient was assessed based on the American Society of Interventional and Therapeutic Neuroradiology/Society of Interventional Radiology grading system, with grades of 0 to 1 on CTA scan at baseline denoting poor collateral state (16).

The primary outcome was functional independence at 90 days (defined as mRS 0–2). Secondary outcomes included early neurological improvement, successful reperfusion following the MTE procedure, any ICH complications, and 90-day mortality. Early neurological improvement was defined as a reduction of at least eight points in the NIHSS score or an NIHSS score of 0 or 1 at 72 h after hospitalization (6). Recanalization rates were assessed before thrombectomy and immediately after MTE reperfusion, according to the modified Thrombolysis in Cerebral Infarction scale (mTICI) score (17). An mTICI score of 2b or three was considered as complete reperfusion. Where available, information on the collateral state was obtained from the medical records. The overall state of collaterals for each patient was assessed based on the American Society of Interventional and Therapeutic Neuroradiology/Society of Interventional Radiology grading system, with grades of 0 to 1 on CTA scan at baseline denoting poor collateral state (16). As the safety outcome, hemorrhagic complications after reperfusion therapy included any ICH, subarachnoid hemorrhage, and other forms of hemorrhage within the cranium identified on brain imaging, or reported by a clinician while in the hospital (6). A sub-parameter of safety outcome was symptomatic cerebral hemorrhage (sICH). An sICH was defined as any hemorrhage identified as the predominant cause of the neurological deterioration (indicated by an NIHSS score that was higher by ≥4 points than the value at baseline or the lowest value in the first 7 days or any hemorrhage leading to death) (6). In addition, the hemorrhage must be classified as parenchymal intracerebral hemorrhage type 2 (dense hematoma >30% of the infarcted area with substantial space-occupying effect or as any hemorrhagic lesion outside the infarcted area) (6).

### Statistical Analysis

All the data analyses were performed using SPSS software, version 21.0 (IBM, Co., Armonk, NY, USA). Parameters were presented as N (%), mean ± standard deviation, or median (25 and75th percentile). Differences between the two groups were assessed using the Pearson correlation test for categorical variables and the Student *t*-test or Mann–Whitney *U*-test, where relevant, for continuous variables. The Fisher exact test was applied for continuous variables when the number of observations was <5. A two-sided *p*-value < 0.05 was considered statistically significant. Multivariable logistic regression models, accounting for potential confounding factors (age, sex, and where possible together with sufficient cases, the variables that showed a *p*-value <0.1), were used to compare clinical outcomes and hemorrhagic complications between the two groups (low- vs. standard-dose alteplase) following bridging therapy. We also performed sensitivity analyses that included some relevant covariates not meeting the criteria for being confounders as specified above to see if these factors impacted different outcomes.

## Results

In this study, we included 107 patients (63% males; mean age: 63 years) who were treated with intravenous alteplase prior to MTE. They were divided into two groups according to the bridging therapy they received: 73 were treated with a lower dose of intravenous alteplase (0.6 mg/kg) and MTE between 2017 and 2018, and 34 were treated with a standard dose of intravenous alteplase (0.9 mg/kg) and MTE in 2019. Table 1 shows that there were some differences in baseline demographic and clinical characteristics between the two groups. Compared to the standard-dose group, those with low-dose alteplase before MTE were more likely to have a more severe stroke, indicated by greater initial NIHSS mean score (median difference: 2 points; *p* < 0.001), and longer onset to hospital arrival time [mean difference (MD): 26.6 min; *p* = 0.005; Table 1]. Conversely, door to needle and needle to groin times for the standard-dose group, compared to the low-dose group, were about 6 min (*p* = 0.094) and 12 min (*p* = 0.042; Table 1), respectively. There were no significant differences between the two groups with regards to the groin to recanalization time, ischemic stroke subtype (Table 1), the ASPECTS, and involved vessels in cerebral angiography (Table 2). The solitaire device was used more frequently (*p* < 0.001) in the low-dose group (78.1%) than in the standard-dose group (26.5%), while the use of the solumbra technique was more common in the standard-dose than the low-dose group (38.2 and 5.5%, respectively; *p* < 0.001). However, there were no significant differences between the two groups in successful recanalization rates (mTICI score 2b−3) before thrombectomy (high-dose 8.8 vs. low-dose 1.4%; *p* = 0.520; Table 2) and after thrombectomy (97.1 and 95.9%; *p* = 0.922; Table 3).

**Table 1:** Baseline demographic and clinical characteristics.

	**0.9 mg/kg + MTE** **(*N* = 34)**	**0.6 mg/kg + MTE** **(*N* = 73)**	***p*-value**
Characteristics	n (%)^*^or mean ± SD	n (%)^*^or mean ± SD	
Sex, males	21 (61.8)	46 (63.0%)	0.901
Age (years)	63.6 ± 11.8	62.4 ± 11.4	0.606
Body weight prior to alteplase (kg)	55.8 ± 7.8	56.3 ± 9.1	0.776
Systolic blood pressure (mmHg)	139.5 ± 17.9	137.8 ± 20.7	0.685
Diastolic blood pressure (mmHg)	80.6 ± 9.4	79.5 ± 9.7	0.579
**Comorbidities**			
Hypertension	15 (44.1)	19 (26.0)	0.076
Atrial fibrillation	2 (5.9)	9 (12.3)	0.497
Previous ischemic stroke	0 (0)	4 (5.5)	0.305
Diabetes mellitus	3 (8.8)	6 (8.2)	0.999
Hypercholesterolemia	2 (5.9)	2 (2.7)	0.590
Current smoker	11 (32.4)	18 (24.7)	0.485
**Pre-stroke medications**			
Anticoagulation	1 (2.9)	0 (0)	0.318
Antiplatelet agents	0 (0)	0 (0)	
**Pre-stroke mRS**			
mRS = 0	33 (97.1)	73 (100)	0.318
mRS = 1	1 (2.9)	0 (0)	
Baseline NIHSS score, mean (interquartile range)	14 (11–16)	16 (14–19)	**0.001**
Onset to hospital arrival (minute)	103.2± 45.9	129.8 ± 43.4	**0.005**
Onset to needle time (minute)	157.1± 43.6	175.6 ± 43.3	**0.043**
**Onset to needle time, category**			
<3 h	22 (64.7)	42 (57.5)	0.531
3–4.5 h	12 (35.3)	31 (42.5)	
Onset to groin puncture (minute)	202.2 ± 53.8	206.3 ± 47.9	0.699
Intracranial atherosclerosis	20 (58.8)	46 (63.0)	0.678
Need of placement of permanent intracranial stent during MTE to achieve recanalization	5 (14.7)	0 (0)	**0.003**
**Subtype by TOAST classification**			
Large artery disease	17 (50.0)	42 (57.5)	0.466
Cardioembolism	10 (29.4)	20 (27.4)	0.829
Undetermined	7 (20.6)	11 (15.1)	0.477

* *Otherwise indicated. Bold denotes statistically significant results. MTE, mechanical thrombectomy; TOAST, trial of ORG 10172 in acute stroke treatment; NIHSS, national institutes of health stroke scale; mRS, modified rankin scale*.

**Table 2:** Baseline characteristics of acute ischemic stroke on cerebral angiography.

	**0.9 mg/kg + MTE (*N* = 34)**	**0.6 mg/kg + MTE (*N* = 73)**	***p*-value**
Characteristics	n (%)^*^	n (%)^*^	
ASPECTS, median (interquartile range)	8 (7–9)	8 (7–9)	0.754
**Involved vessel (occlusion site)**			
Internal carotid artery	9 (26.5)	16 (21.9)	0.604
Tandem	4 (11.8)	11 (15.1)	0.647
M1 segment of MCA	16 (47.1)	36 (49.3)	0.828
M2 segment of MCA	5 (14.7)	10 (13.7)	0.889
Door to needle (minute)	53.9 ± 25.1	45.8 ± 13.5	0.094
Door to groin (minute)	98.5 ± 35.5	76.5 ± 20.1	**0.002**
Needle to groin time (minute)	42.9 ± 32.4	30.7 ± 14.9	**0.042**
**Mechanical thrombectomy technique**			
Solitaire	9 (26.5)	57 (78.1)	**<0.001**
Penumbra	8 (23.5)	11 (15.1)	0.284
Solumbra	13 (38.2)	4 (5.5)	**<0.001**
No device	4 (11.8)	1 (1.4)	**0.034**
**Collateral status, grade**			
0–1 (poor collateral)	14 (41.2)	0 (0)	**<0.001**
>1	11 (32.4)	34 (46.6)	
Missing data	9 (26.5)	39 (53.4)	
Successful reperfusion before thrombectomy	3 (8.8%)	1 (1.4%)	0.520
Number of attempts to achieve recanalization	1 (1–2)	2 (1–2)	0.075
Recanalization achieved ≤ 24 h	27 (79.4)	61 (83.6)	0.601
Groin to recanalization time (minute)	40.4 ± 28.6	43.0 ± 25.8	0.403
Onset to recanalization time (minute)	242.7 ± 65.3	249.3 ± 54.6	0.587

* *Otherwise indicated. Bold denotes statistically significant results. MTE, mechanical thrombectomy; ASPECTS, alberta stroke programme early CT score; mTICI, modified thrombolysis in cerebral infarction scale; MCA, middle cerebral artery*.

**Table 3:** Outcomes and complications of low-dose and standard-dose intravenous alteplase bridging with thrombectomy.

**Variable**	**0.9 mg/kg (*N* = 34) *n* (%)**	**0.6 mg/kg (*N* = 73) *n* (%)**	***p*-value^†^**	**Unadjusted OR**	**Adjusted OR (95% CI), model A^‡^**	**Adjusted OR (95% CI), model B^‡^**
**Primary outcome**						
90-day functional independence (mRS ≤ 2)	22 (64.8)	50 (68.5)	0.053	0.84 (0.36–1.99)	1.02 (0.29–3.52)^a^	1.12 (0.31–4.13)^b^
**Secondary outcomes**						
Early neurologic improvement^*^	14 (41.2)	46 (63.0)	0.204	2.43 (1.06; 5.59)	1.89 (0.54–6.66)^a^	1.91 (0.52–6.98)^c^
Successful recanalization (TICI score 2b−3)	33 (97.1)	70 (95.9)	0.922	0.71 (0.07–7.06)	1.18 (0.10–13.9)^d^	1.24 (0.08–20.5)^e^
Any ICH while in hospital	11 (32.4)	19 (26.0)	0.083	0.74 (0.30–1.79)	0.58 (0.18–1.84)^d^	0.49 (0.15–1.65)^f^
sICH	4 (11.8)	4 (5.5)	0.085	0.43 (0.10–1.85)	0.30 (0.04–2.41)^d^	0.16 (0.02–1.46)^f^
90-day mortality	3 (8.8)	2 (2.7)	0.053	0.29 (0.05–1.83)	0.16 (0.01–2.47)^d^	0.11 (0.01–1.18)^b^

*MTE, mechanical thrombectomy; mRS, modified rankin scale; ICH, intracranial hemorrhage; sICH, symtomatic ICH; NIHSS, national institutes of health stroke scale; OR, odds ratio; NA, not applicable due to insufficient cases*.

* Early neurologic improvement defined as a reduction of ≥8 points on the NIHSS score, or an NIHSS score of 0 or 1 at 3 days;

† From Chi-square test;

‡ *Model A included age, sex, and the variables that showed a p-value <0.1 (where possible together with sufficient cases) as specified in the Methods section. Model B, as a sensitivity analysis, included the factors not meeting the criteria for inclusion in model A but potentially impacting relevant outcomes*.

a *Adjusted for age, sex, hypertension, initial NIHSS, onset to needle time, needle to groin time, and MTE technique*.

b *Adjusted for age, sex, hypertension, initial NIHSS, onset to needle time, needle to groin time, groin to recanalization time, MTE technique*.

c *Adjusted for age, sex, hypertension, initial NIHSS, onset to needle time, needle to groin time, groin to recanalization time, MTE technique, recanalization ≤ 24 h*.

d *Adjusted for age, sex, hypertension, initial NIHSS, onset to needle time, and needle to groin time*.

e *Adjusted for age, sex, hypertension, initial NIHSS, onset to needle time, needle to groin time, groin to recanalization time, successful reperfusion before thrombectomy, and number of attempts to achieve recanalization*.

f *Adjusted for age, sex, hypertension, initial NIHSS, onset to needle time, needle to groin time, groin to recanalization time, and number of attempts to achieve recanalization*.

The factors that met our criteria for being included in the multivariable models were age, sex, history of hypertension, initial NIHSS, onset to needle time, needle to groin time, and where applicable, the MTE technique was used. After accounting for potential confounding factors (Table 3), it was found that early neurologic improvement, recanalization, 90-day mortality, and functional outcome at 90 days were not statistically significant between the two groups. Those receiving low-dose alteplase before MTE had lower odds of having ICH complications compared to the standard-dose group, but the differences were not statistically significant. The findings were consistent with those from sensitivity analyses accounting for some relevant covariates not meeting the criteria for being confounders (i.e., groin to recanalization time, successful reperfusion before thrombectomy, and a number of attempts to achieve recanalization; Table 3).

## Discussions

Our single-center retrospective study showed no statistically significant differences in clinical outcomes and ICH complications between two groups of LVO-AIS patients treated with low-dose vs. standard-dose bridging alteplase before MTE, after accounting for confounding factors. The findings are consistent with those from the Korean (18)and Taiwanese contexts (19), noting the limited number of cases with bridging therapy (Taiwan, *N* = 42; Korea; *N* = 64; the present study; *N* = 107).

For both groups, MTE was performed immediately following thrombolysis and we continued alteplase infusion during thrombectomy, even with those who achieved successful recanalization. High recanalization rates (>95%) presented in this study are consistent with previous studies conducted in Asian populations. In the SKIP trial conducted in Japan, the rate of successful reperfusion was 92% among those receiving low-dose alteplase bridging with MTE (2, 20). Similar high recanalization rates were observed in the Korean ENCHANTED study on a subset of patients receiving alteplase bridging with MTE (low-dose IVT: 85% vs. standard-dose IVT: 76%) (18) and the Taiwanese study (low-dose IVT: 100% vs. standard-dose IVT: 69%) (19). In a study conducted in Southern Vietnam, among those with standard-dose alteplase bridging therapy, the recanalization rate was approximately 88% (onset to groin time ~270 min) (21). Our study findings showed no significant time delays occurred between drip and groin puncture time (~40 min; onset to groin time: up to 206 min), which may contribute to the high recanalization rates in both arms, despite a relatively high rate of large artery disease etiology presented in the study (~50%). There is a possibility of selection bias in the study. There were significant delays in stroke onset to hospital arrival time whereby about 70% of those with stroke admitted to our stroke unit are not eligible for IVT therapy. In our stroke center, MTE was performed in 160 ischemic stroke patients in 2017, 180 in 2018, 230 in 2019, but most of them were provided with thrombectomy procedures, either within 4.5–8 h of stroke onset or within 4.5 h, but they also had contraindications for intravenous thrombolysis (IVT). These patients were therefore excluded from the study, which had a focus on comparing low-dose vs. standard-dose intravenous alteplase (≤4.5 h) prior to MTE (≤ 6 h).

Our study has several limitations and strengths. This is a single-center retrospective study that included patients receiving either standard- or low-dose alteplase prior to MTE from two different time periods. There could be a risk of selection bias due to the lack of randomization. We attempted to account for the differences in patient characteristics and clinical factors between two groups in our data analyses. However, residual confounding is likely due to poorly measured (e.g., collateral status) or unmeasured confounding factors (e.g., thrombus time) in the study. The sample size was small, which limited our study power particularly for comparing secondary outcomes (e.g., ICH complications) among the two groups of patients. We were also unable to perform sensitivity analyses using alternative statistical methods, such as the propensity score matching approach. To our knowledge, this is one of the rare three studies (18, 19), all based on Asian populations, and the study with the largest sample among the three to compare low-dose and standard-dose in bridging therapy to date. Given potential racial and ethnic differences in patient characteristics and outcomes after reperfusion therapy (e.g., higher risk of ICH and intracranial atherosclerosis), the conclusion of the study can only be applicable to an Asian population. Our study is the first to report the outcomes among Vietnamese patients with different doses of intravenous alteplase prior to MTE using data from a high-volume stroke center with no significant delays in door to groin times and higher reperfusion rate. However, the findings should be interpreted in the context of selected high-volume stroke centers in Asia whereby only a small proportion of patients were eligible for endovascular treatment due to delays in stroke onset to hospital arrival time.

The study findings show no differences in clinical outcomes and ICH complications between low-dose vs. standard-dose bridging alteplase before MTE. The number of treated patients was too low to show non-inferiority of low-dose treatment. The approach of treating low-dose alteplase patients with LVO-AIS may prove beneficial by future studies for cases presenting directly to a thrombectomy-capable stroke center and to Asian populations with higher bleeding risks. Larger randomized trials are needed to demonstrate whether a low alteplase dose retains the same efficacy as the standard dose. The role of tenecteplase (TNK) in bridging therapy could also prove to improve clinical outcomes in patients requiring bridging therapy. According to the Tenecteplase vs. Alteplase before Endovascular Therapy for Ischemic Stroke (EXTEND-IA TNK) trial (22), TNK before thrombectomy demonstrated at least non-inferior to alteplase in restoring perfusion in the territory of a proximal cerebral-artery occlusion and functional outcome than alteplase among patients with ischemic stroke treated within 4.5 h after symptom onset. The Ministry of Health in Vietnam has only approved the use of TNK for patients with acute myocardial infarction. The possible superiority of TNK compared to alteplase suggests that the newer generation of thrombolytic drugs (e.g., TNK) are promisingly alternative thrombolytic for cases with LVO-AIS in future practice, but further clinical trials are required.

## Data Availability Statement

The raw data supporting the conclusions of this article will be made available by the authors, without undue reservation.

## Ethics Statement

This retrospective study was approved by the Ethics Committee of Hanoi Medical University, No 187/HÐÐÐÐHYHN on February 20, 2016.

## Author Contributions

All authors made substantial contributions to the study concept and design, acquisition of data, or data analysis, and interpretation of data. The authors also took part in drafting the article, and/or revising the manuscript critically for important intellectual content, read and gave final approval of the version to be published, and agreed to be accountable for all aspects of the work.

## Conflict of Interest

The authors declare that the research was conducted in the absence of any commercial or financial relationships that could be construed as a potential conflict of interest.
